# CUPAC – The Coventry University public road dataset for automated cars

**DOI:** 10.1016/j.dib.2019.104950

**Published:** 2019-12-07

**Authors:** Yannik Weber, Stratis Kanarachos

**Affiliations:** Research Institute Future Transport and Cities, Coventry University, Priory Street, CV1 5FB, United Kingdom

**Keywords:** Public road data, Automated vehicles, Computer vision, Vehicle dynamics, SLAM, Target state estimation, Road anomalies

## Abstract

This article presents a dataset recorded with a sensor-equipped research vehicle on public roads in the city of Coventry in the United Kingdom. The sensor suite includes a monocular-, infrared- and smartphone-camera, as well as a LiDAR unit, GPS receiver, smartphone sensors and vehicle CAN bus data logger. Data were collected by day and night in a variety of traffic, weather and road surface conditions with a focus on the correlation between vehicle dynamics and the environmental perception process of automated vehicles.

**Table:** Specifications Table

Subject	Computer Vision and Pattern Recognition
Specific subject area	Environmental Perception of Automated Vehicles
Type of data	Matlab Structure (CAN-Bus, Smartphone Sensors) Image (Cameras) Point Cloud (Laser Scanner)
How data were acquired	• Racelogic VBOX Video Monocular Camera (1920 × 1080 resolution/30 fps) [1] • Racelogic VBOX Video HD2 CAN-Bus Data Logger (10 Hz) • Racelogic VBOX Video HD2 GPS Antenna (10 Hz) • Velodyne VLP-16 Laser Scanner (600 rpm/10 Hz) [2] • FLIR One Pro Infrared Camera (1080 × 1440 resolution (160 × 120 Thermal resolution)/8–9 fps) [3] • Samsung Galaxy S8 Smartphone Camera (1080 × 720 resolution/30 fps) • Samsung Galaxy S8 Smartphone Sensors with AndroSensor Application (10 Hz)
Data format	Raw
Parameters for data collection	Data was collected with a sensor-equipped vehicle while driving naturalistically on public roads. In particular: Ego-vehicle position, speed, and acceleration. Stationary and moving obstacle detection using visual and infrared cameras, and LIDAR sensors.
Description of data collection	Measurements were conducted by driving naturalistically during day and night in various traffic, weather and road surface conditions.
Data source location	City/Town/Region: Coventry Country: United Kingdom Latitude and longitude (and GPS coordinates) for collected samples/data: GPS driving routes part of dataset
Data accessibility	Repository name: Zenodo Data identification number: 3383693 Direct URL to data: https://doi.org/10.5281/zenodo.3383693

**Table:** 

**Value of the Data** • To the authors' best knowledge this is the first dataset recorded with a focus on obstacle detection and tracking under vehicle vertical dynamics excitation caused by road anomalies such as road bumps and potholes. • The naturalistic driving studies were conducted in an urban environment, specifically Coventry city in the U.K. Driving takes place on the left side of the road. Furthermore, it is the first presentation of a low-cost infrared sensor in combination with two different monocular cameras and a LiDAR unit. • The dataset can be used by research institutions, manufacturers and suppliers as well as independent developers in the development and testing process of automated driving functions. • The data is beneficial to estimate the influence of vehicle dynamics on the environmental perception process of automated vehicles. It is also useful to research the added value of low-cost infrared sensing in detecting road objects. • Additionally, the data can serve as a benchmark to validate environment-modelling functions of automated vehicles in challenging vehicle dynamics test cases.

## 1 Data

The included files consist of recorded data from four different days of public road driving trials, divided into the sets of alpha, bravo, charlie and delta. Following table presents the specifications of each scenario including the traffic volume of driving (Traff. D.) and parked vehicles (Traff. P.), the weather condition (Sunny, Cloudy, Clear), the time of the day and driving environment key features of each trial. The selection of features also reflects the parametrization of driving scenarios in the simulation software IPG Carmaker. While alpha, bravo and charlie road trials include the monocular and infrared camera, LiDAR scanner, GPS receiver and CAN-bus data logger, delta road trials also consist of additional smartphone's camera and sensors measurement data. All features are summarised in Table 1.

**Table 1:** General features of the data set.

No	Length [min]	Traff. D.	Traff. P.	Weather	Time	Environmental features
a1	6.4	High	High	Cloudy	Afternoon	Inner-city, Parking lot
a2	8.3	Low	High	Cloudy	Afternoon	Parking lot, Country road
a3	8.3	High	Low	Cloudy	Afternoon	Inner-city, Country road
a4	8.3	High	Medium	Cloudy	Afternoon	Inner-city
b1	6.28	Medium	Medium	Sunny	Noon	Inner-city
b2	16.92	Low	High	Sunny	Noon	Residential area, Road bumps
b3	25.38	Medium	High	Sunny	Noon	Residential area, Road bumps, Inner-city
c1	4.86	Medium	Medium	Clear	Evening	Country road, Parking lot
c2	11.54	Low	Low	Clear	Night	Inner-city, Country road
c3	22.97	Medium	High	Clear	Night	Parking lot, Highway, Residential area, Road bumps
c4	11.44	Low	High	Clear	Night	Residential area, Road bumps, Highway, Inner-city
d1	8.3	Low	Low	Clear	Night	Highway, Residential area
d2	8.3	Low	High	Clear	Night	Residential area, Road bumps
d3	8.3	Low	High	Clear	Night	Residential area, Road bumps, Highway

The sensor streams were manually synchronised by matching the first and last frames of each sensor in every scenario. One remark must be made here: As the infrared camera's frame rate is not consistently constant, there might be delays between the infrared and the other sensor streams. A consistent time stamp for the infrared camera will be subject to future updates to the dataset. For more information on the synchronisation procedure, the interested reader should feel free to get in touch with the corresponding author.

To ensure ethically correct data handling, the dataset complies with the General Data Protection Regulation (GDPR). Every video sequence collected by all the three deployed cameras was scanned manually for vehicle number plates and people's faces. The features were then marked with the Matlab Ground Truth Labeller application and tracked with a point tracker [4]. Afterwards, every sequence was analysed again to mitigate human errors. Marked regions were blurred with a Gaussian filter.

Part of the dataset are also the camera calibration parameters and errors. These include in particular the image size, radial distortion, world points, translation vectors, reprojection errors, rotation vectors, intrinsic matrix, focal length, principal point, mean reprojection error, reprojected points and rotation matrices. The error files include the intrinsics and extrinsics errors.

**Fig. 1:**
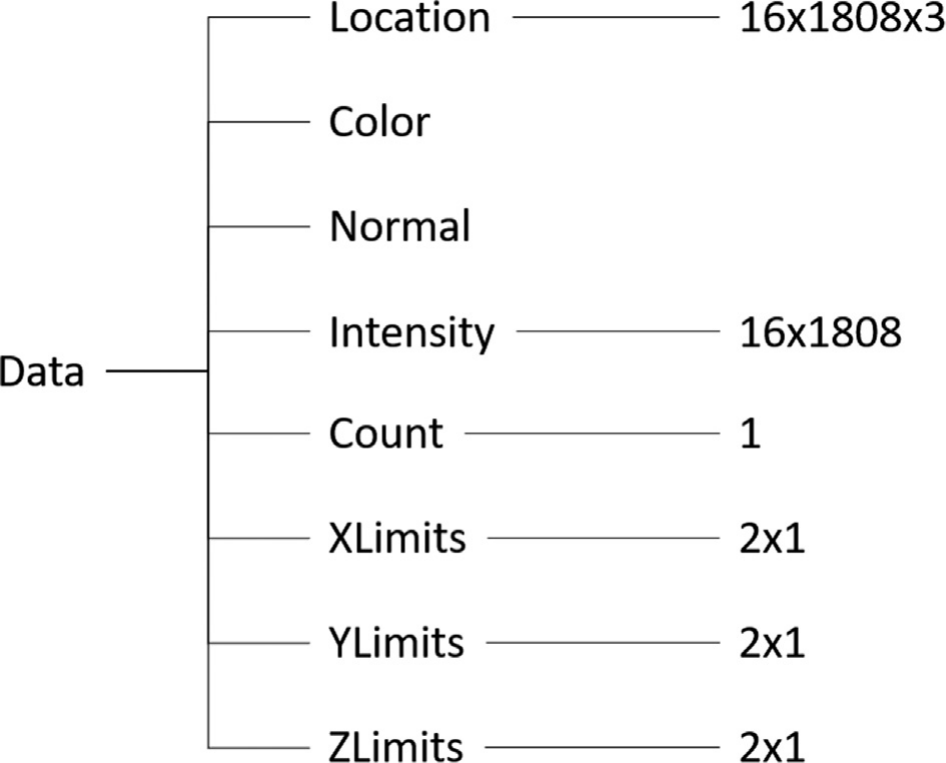
Structure of the acquired LiDAR point cloud data.

The LiDAR point cloud information is stored in Matlab point cloud objects of the appearance illustrated in following Fig. 1. This information can be used to recreate the real-world trials in a simulation environment like e.g. IPG CarMaker.

The location branch is providing the 3D-position of each of the 16 laser beams with intensity values in a separate column. Count sums up the number of collected laser beams (in this case 16 times 1808) per point cloud with minimum and maximum X-, Y- and Z-values added to the data object. A separate LiDAR data object was created for every time stamp in steps of 10 Hz.

**Fig. 2:**
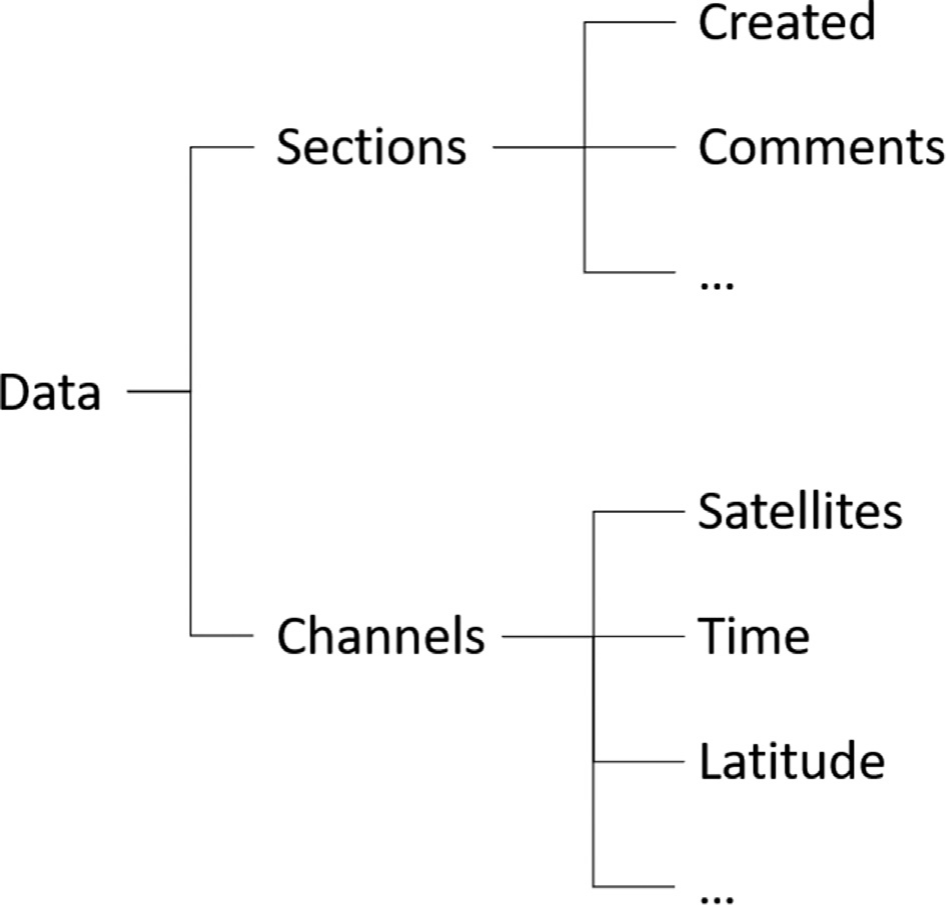
Structure of the acquired CAN-bus data.

The acquired CAN-bus data consists of the following information: Number of visible GPS satellites, time, latitude, longitude, velocity, heading, height, vertical velocity, steering angle, all wheel speeds, yaw rate, vehicle speed, longitudinal acceleration, lateral acceleration, handbrake, gear requested, gear, engine speed, coolant temperature, clutch position, brake pressure, brake position, battery voltage, air temperature and accelerator pedal position. The data is stored in Matlab structures whose layout is presented in Fig. 2.

In addition to previously introduced CAN-bus data saved in different channels, more generic information on the recording session is given in the sections path of the structure. The channels themselves consist of one-dimensional column vectors sorted in 10 Hz frequency steps. In total, we collected 19 GB of infrared, 57 GB of LiDAR, 39 GB of monocular and 2.3 GB of smartphone data.

## 2 Experimental design, materials, and methods

**Fig. 3:**
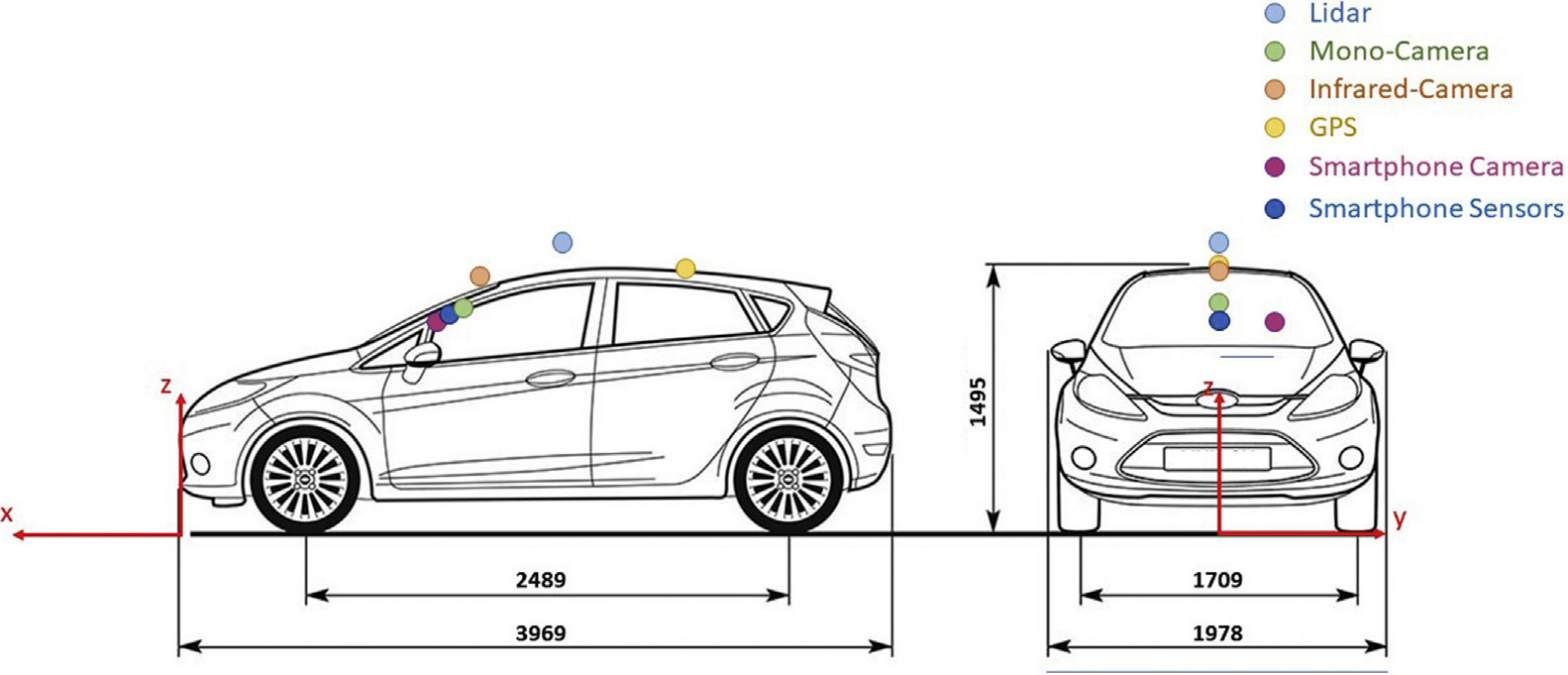
Vehicle dimensions and sensor positions [5].

The LiDAR unit, infrared camera and GPS receiver were mounted on the outside of the research vehicle. The residual sensors are placed inside the car. The exact sensor positions are summarised in following Table 2 and refer to the vehicle coordinate system presented in Fig. 3. The presented tables and figures in this chapter are not part of the data set itself but serve as additional information required for setting up e.g. target state estimation, localisation or mapping algorithms.

**Table 2:** Sensor positions in vehicle coordinates.

Sensor	X [mm]	Y [mm]	Z [mm]
Mono Camera	1570	0	1260
Infrared Camera	1660	0	1430
LiDAR	2120	0	1630
Smartphone Camera	1420	310	1150
Smartphone Sensors	1490	0	1180

**Fig. 4:**
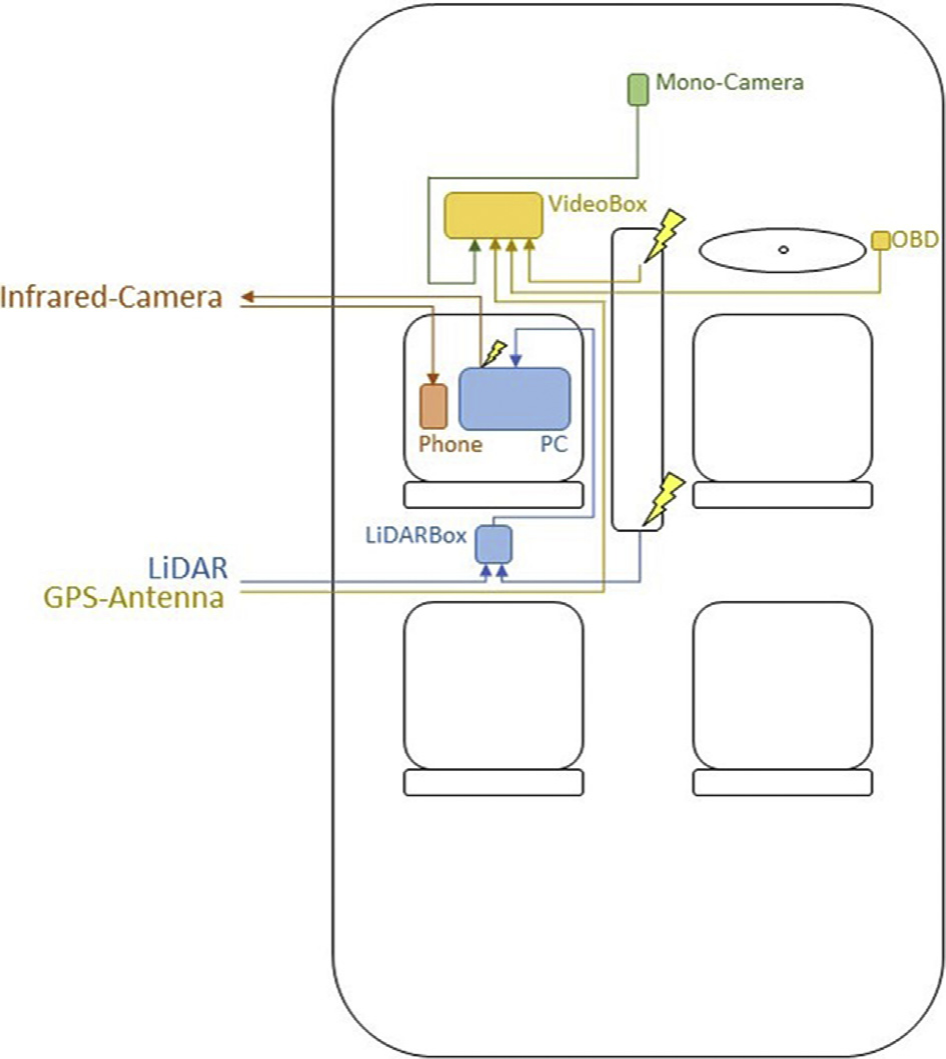
Interior sensor wiring.

The sensor wiring inside the vehicle is shown in Fig. 4 with power supply highlighted as yellow lightnings.

**Fig. 5:**
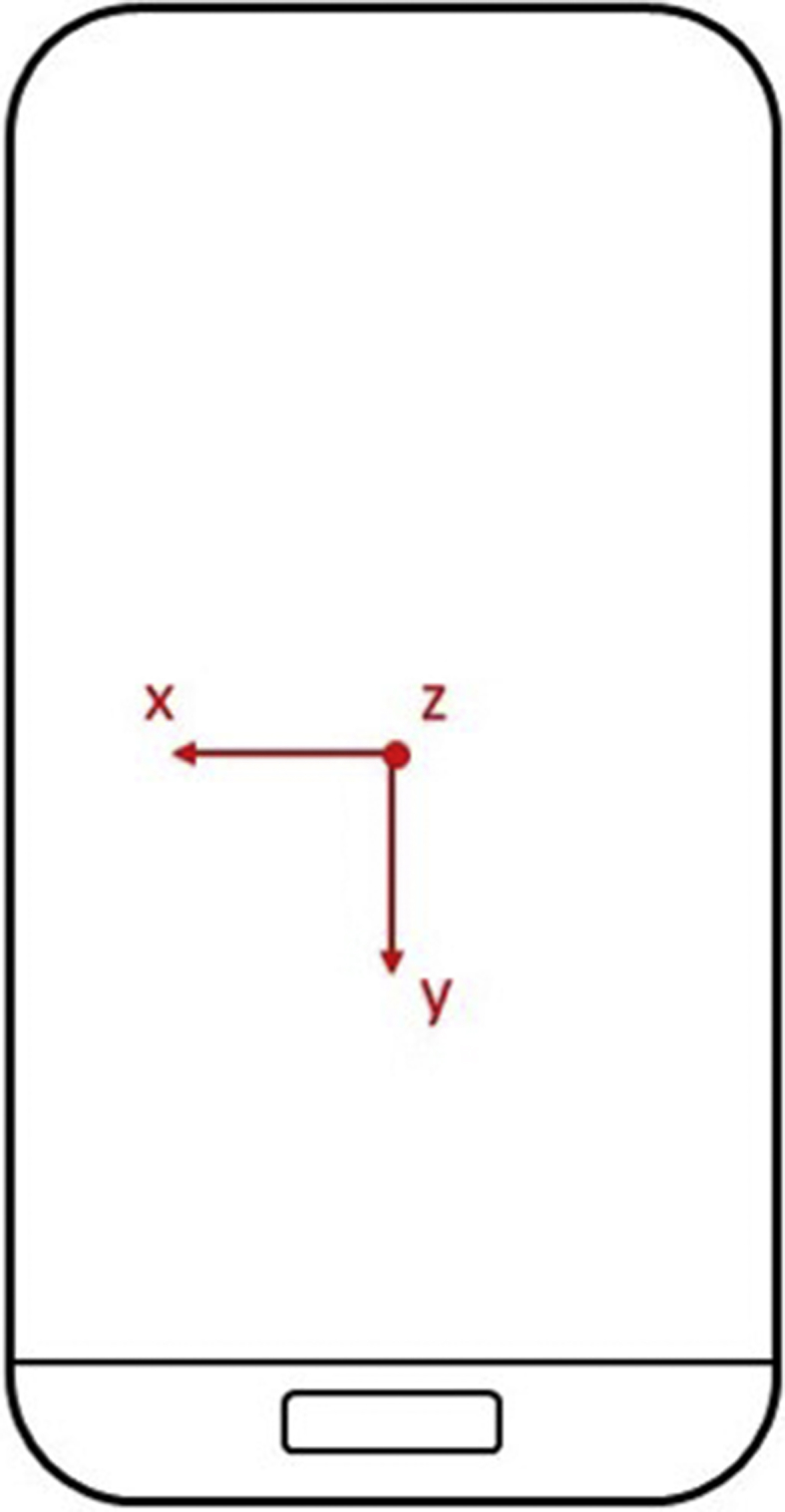
Smartphone accelerometer coordinate system.

The smartphone sensor coordinate system is displayed in following Fig. 5.

To generate the camera calibration and error files, the Matlab Camera Calibrator application was used [6]. With the monocular and smartphone camera, pictures of a calibration pattern (checkerboard) were taken in different angles. With help of these images, aforementioned camera intrinsic parameters were extracted according to Refs. [7,8].

For data analysis we highly recommend Matlab in combination with additional packages like the ‘Statistics and Machine Learning Toolbox’, ‘Deep Learning Toolbox’, ‘Computer Vision Toolbox’ or the ‘Automated Driving Toolbox’. These libraries allow the visualisation of all attached data files and furthermore the fusion of e.g. camera and LiDAR data.
